# HER2 in Metastatic Colorectal Cancer: Pathology, Somatic Alterations, and Perspectives for Novel Therapeutic Schemes

**DOI:** 10.3390/life12091403

**Published:** 2022-09-09

**Authors:** Mariia Ivanova, Konstantinos Venetis, Elena Guerini-Rocco, Luca Bottiglieri, Mauro Giuseppe Mastropasqua, Ornella Garrone, Nicola Fusco, Michele Ghidini

**Affiliations:** 1 Division of Pathology, IEO, European Institute of Oncology IRCCS, University of Milan, Via Giuseppe Ripamonti 435, 20141 Milan, Italy; 2 Department of Emergency and Organ Transplantation, School of Medicine, University of Bari “Aldo Moro”, Piazza G Cesare, 11, 70124 Bari, Italy; 3 Medical Oncology Unit, Fondazione IRCCS Ca’ Granda Ospedale Maggiore Policlinico, 20122 Milan, Italy

**Keywords:** HER2, colorectal cancer, pathology, biomarkers, targeted therapy

## Abstract

**Simple Summary:**

In the current clinical practice, HER2 status is tested in breast and gastroesophageal cancers to select patients eligible for anti-HER2 treatment. However, HER2 is an emerging biomarker in colorectal cancer (CRC), one of the big killers in oncology. The most frequent types of HER2 alterations in CRC include gene amplification and mutations and often involve protein overexpression. In this review, we discuss the current knowledge of HER2 testing in CRC and the immediate future perspectives for HER2 targeting in the metastatic setting.

**Abstract:**

HER2 is an emerging biomarker in colorectal cancer (CRC). This oncogene plays an essential role in regulating cell proliferation, differentiation, migration, and, more in general, tumorigenesis and tumor progression. The most frequent types of HER2 alterations in CRC include gene amplification and missense mutations in 7–8% of CRC, often being mirrored by HER2 protein overexpression, representing founder events in solid tumors, including CRC. There are currently no approved HER2-targeted therapy guidelines for CRC; however, several studies have shown that HER2 can be effectively targeted in meta-static CRC settings. In this review, we discuss the current knowledge of HER2 testing in CRC and the immediate future perspectives for HER2 targeting in the metastatic setting.

## 1. Introduction

Human epidermal growth factor receptor 2 (*HER2*) is a proto-oncogene encoding for a transmembrane glycoprotein with a tyrosine kinase activity, a member of the ErbB receptor tyrosine kinases family, and one of the epidermal growth factor receptors (EGFRs) [1,2]. *HER2* plays an essential role in normal biological and oncogenic processes, regulating cell proliferation, differentiation, and migration via numerous signaling pathways, such as mitogen-activated protein kinase/extracellular signal-regulated kinases (MAPK/ERK) and phosphoinositide 3 kinase (PI3K)/Akt/mammalian target of rapamycin (mTOR) [1,2]. The schematic overview of *HER2* pathways is represented in Supplementary Figure S1. Alterations of *HER2* include gene amplification and missense mutations and often lead to protein overexpression [3]. These types of molecular aberrations are considered founder events in tumorigenesis and tumor progression because of uncontrolled cell proliferation, inhibition of apoptosis, and migration [1,4,5,6].

In the current clinical practice, HER2 status is tested in breast and gastroesophageal cancers to select patients eligible for anti-HER2 treatment [5]. However, alterations in HER2 have been documented in a plethora of other solid tumors, including colorectal cancer (CRC) [2,3,4,5,6,7]. This tumor type is the second leading cause of cancer-associated deaths worldwide in both sexes [8]. In CRC, the frequency of HER2 overexpression is 5–6% with somatic *HER2* gene alterations, including amplifications, reported in ~7% of patients [4,6,9,10,11]. *HER2* mutations in colonic epithelial cells have been shown to be indicative of HER2 signaling pathway activations, promote independent cell growth, and potentially acquire resistance to EGFR-targeted therapies, subjecting patients to a worse prognosis [2,4,6,9,10,11,12]. There are currently no approved HER2-targeted therapy guidelines for CRC; however, several studies have shown that HER2 can be effectively targeted in metastatic CRC settings [2,12,13,14].

In this review, we discuss the current knowledge of HER2 testing in CRC and its significance in both translational research and clinical studies. Particular emphasis will be given to the immediate future perspectives for HER2 targeting in patients with CRC in the metastatic setting (mCRC).

## 2. HER2 Targeting in Metastatic Colorectal Cancer

The administration of anti-HER2 drugs (e.g., trastuzumab, pertuzumab) is presently a standard of care in HER2-positive (i.e., score 3+ by immunohistochemistry (IHC) or 2+/in situ hybridization (ISH)-positive) breast and gastric cancer [15,16]. For CRC, HER2 first emerged as a negative predictive biomarker. Amplification or overexpression of HER2 was associated with a lack of response to anti-EGFR treatment [17].

The novel antibody–drug conjugate trastuzumab deruxtecan (T-DXd) has been approved by the United States Food and Drug Administration (FDA) for HER2-low (i.e., score 1+ or 2+/ISH-negative) breast cancers [18], and it is currently under investigation in histology-agnostic settings in the DESTINY-PanTumor02 study (NCT04482309). The first clinical trials using trastuzumab in mCRC evaluated the combination of this monoclonal antibody with chemotherapy. Clark et al. assessed the association of FOLFOX + trastuzumab in the second- or third-line treatment of *HER2*-positive mCRC. A total of 5 patients out of 21 (24%) had a partial (PR) or complete response (CR), with a median duration of response of 4.5 months [19]. Another phase II study assessed the combination of trastuzumab and irinotecan in *HER2*-positive mCRC pretreated with one line of therapy. Objective responses were recorded in five patients (71%), and these were maintained for at least 6 months [20].

Subsequent studies evaluated different strategies of an *HER2* dual blockade with significant results. HERACLES-A tested the combination of trastuzumab and lapatinib in patients with *KRAS* exon-2 wild-type (WT) mCRC refractory to standard treatment and with HER2 amplification and/or overexpression. A total of 914 patients were screened, of whom 48 (5%) had *HER2*-positive diseases [14,21]. The long-term clinical results at a follow-up of 6.7 years reported an overall response rate (ORR) of 28% with one CR and eight PRs, a disease control rate (DCR) of 69%, median progression-free survival (mPFS) of 4.7 months, and median overall survival (OS) of 10 months for 32 treated patients. Two of them (6%) reported a grade 3 decrease in left ventricular ejection fraction, while fatigue was registered in five cases (16%) [22]. Differently, HERACLES-B tested the combination of pertuzumab and the antibody–drug conjugate trastuzumab emtansine (TDM-1) in *RAS* and *BRAF* WT and *HER2*-positive mCRC refractory to standard therapies. The primary endpoint was not reached, with ORR below the expected rate ≥30% (9.7%). Stable disease (SD) was seen in 21 patients (67.7%), and the DCR rate was 77.4%. Treatment was well-tolerated, with two patients suffering from grade (G) 3 thrombocytopenia [23]. A median PFS of 4.2 months was similar to the HERACLES-A study, and patients with a *HER2* 3+ score at immunohistochemistry (IHC) had a significantly higher mPFS compared to *HER2* IHC and fluorescent in situ hybridization (FISH)-amplified tumors (HR: 0.20; 95% confidence interval (CI): 0.07–0.56; *p* = 0.0008) [17]. The MyPathway phase II trial enrolled 57 patients with *HER2*-amplified mCRC receiving a combination of trastuzumab and pertuzumab. One patient had a CR while seventeen (30%) benefited from a PR. On the whole, 18 patients (32%) achieved an objective response, and, in 4 cases, it was longer than 12 months. Treatment was well-tolerated, with the most common adverse events being G 1 or 2 diarrhea, fatigue, and nausea. Patients harboring a *KRAS* mutation had a significantly shorter PFS and OS compared to the *KRAS* WT population (PFS: 1.4 months; 95% CI, 1.2–2.8 months versus 5.3 months; 95% CI: 2.7–6.1 months for mutated and WT, respectively; OS: 8.5 months; 95% CI: 3.9–not estimable versus 14.0 months; 95% CI: 8.0–not estimable for mutated and WT, respectively) [24]. Two other phase II studies (TRIUMPH and TAPUR) evaluated the combination of trastuzumab + pertuzumab. In the first study, 19 patients with *RAS* wt mCRC and *HER2* amplification in tissue samples achieved an ORR of 35% with a complete response and five partial responses. Interestingly, the TRIUMPH trial evaluated *HER2* status on circulating tumor DNA (ctDNA). Similarly, to the patients with *HER2* amplification detected on tissue, 15 patients with ctDNA positivity for *HER2* amplification had an ORR of 33% with one CR and four PRs. With a median follow-up of 5.4 months, mPFS was 4 months [25]. In the TAPUR basket trial, a cohort of 28 patients with *HER2* amplified mCRC was treated; ORR was 14% and DCR for at least 16 weeks was 50% with an mPFS of 3.8 months [26]. A new anti-*HER2* agent, trastuzumab deruxtecan, was tested in the DESTINY-CRC01 phase II trial. This antibody–drug conjugate of a humanized anti-*HER2* antibody with a topoisomerase I inhibitor was tested in *HER2*-positive *RAS-BRAF* WT mCRC progressed on two or more lines of treatment, with the possibility to include patients pretreated with different anti-*HER2* agents. A total of 78 patients were enrolled with 53 placed in cohort A (*HER2* IHC 3+ or 2+ and positive in situ hybridization), a total of 7 in cohort B (IHC 2+ and negative in situ hybridization), and 18 in cohort C (IHC 1+). After a median follow-up of 27.1 weeks, the ORR in group A was 45.3% (95% CI, 31.6–59.6), and patients pretreated with anti-*HER2* agents obtained a high ORR of 43%, as well. Differently, no responses were seen in groups B and C of treatment [12]. At a longer median follow-up of 62.4 weeks with 86 patients treated, the ORR of the group A was confirmed (45.3%). Moreover, DCR was 83%, mPFS 6.9 months, and mOS 15.5 months. Pulmonary toxicity in terms of interstitial lung disease and pneumonitis was recorded in eight patients (9.3%). Two patients died because of grade 5 lung toxicity. Interestingly, trastuzumab deruxtecan was also effective in the group of patients with *RAS* mutation-positive ctDNA [27]. In the ongoing MOUNTAINEER trial, trastuzumab is associated with tucatinib, a tyrosine kinase inhibitor (TKI) of the *HER2* protein. Twenty-six patients with chemorefractory, *RAS* WT, and *HER2*-positive tumors have been treated so far with an ORR of 52.2%, twelve PRs, and six cases of SD. Patients experienced a prolonged median response of 10.4 months, an mPFS of 8.1 months (95% CI, 3.8–not estimable), and a median OS of 18.7 months (95% CI, 12.3–not estimable) [28]. The HER2 FUSCC-G trial is testing the combination of trastuzumab + pyrotinib, an irreversible dual pan-ErbB tyrosine kinase inhibitor (TKI) [29]. A cohort of 11 mCRC *HER2*-positive patients have received this combination so far, with a global ORR of 45.5% and 55.6% in *RAS* WT tumors. With a median follow-up of 17.7 months, mPFS was 7.8 months, while mOS 14.9 months. Patients harboring *KRAS* mutation had poorer outcomes compared to the *KRAS* WT group (PFS, 7.7 versus 9.9 months; *p* = 0.19; OS, 12.4 versus 20.6 months; *p* = 0.021) [30]. Differently, the combination of the anti-*HER2* TKI neratinib and anti-EGFR agent cetuximab was not effective in terms of objective responses. A total of 16 patients with *KRAS*, *NRAS*, *BRAF,* and *PI3KCA* WT tumors were treated; 6 of them (44%) had SD with 5 harboring *HER2* amplification at baseline. G 3adverse events, such as diarrhea, skin rash, and an increased level of transaminases, were registered in 67% of patients [31]. Table 1 lists the main trials with *HER2*-targeted therapies in mCRC that have been conducted so far.

While anti-HER2 therapy in CRC is awaiting approval, many early trials continue, demonstrating promising results [32]. 

Supposing that HER2 may be immunogenic and leads to T-cell activation suggests it is targetable for immunotherapy [33].

Some studies demonstrate the induction of HER2 downregulation in HER2-positive cancer cells with the immune effector cells’ engagement, revealing a new function of immune cells in trastuzumab-mediated antitumor efficacy and probably representing a novel mechanism of action of trastuzumab, predicting active immune effector cells’ recruitment in the tumor microenvironment [34]. 

The acquisition of drug resistance to trastuzumab has been recently explained in HER2-positive gastric cancer by vessel destabilization and activation of the glycolytic pathway inducing 6-phosphofructo-2-kinase (PFKFB3). The inhibition of PFKFB3 in patient-derived xenograft models has significantly diminished tumor proliferation and promoted vessel normalization. It has also been found that PFKFB3 promotes the interleukin-8 coding gene CXCL8 by activating the PI3K/AKT/NFκB p65 pathway, which leads to the idea that PFKFB3 inhibition might be effective in overcoming trastuzumab resistance in HER2-positive cancers [35]. 

HER2-activating mutations are also known for their association with microsatellite instability-high tumors, which has been observed in CRC [36]. With the evolution of immunotherapy, after the US Food and Drug Administration (FDA) approval of pembrolizumab, it has become a practice-changing treatment option for unresectable or mCRC in patients with high microsatellite instability (MSI-H) or mismatched repair deficiency (dMMR) [37].

In the metastatic settings, however, only a small proportion of CRC patients responded to immune checkpoint therapy, despite positive results in some phase I trials, and all these tumors were MSI-H/dMMR and had a high tumor mutation burden [38]. While tumor mutational burden has been associated with the immune checkpoint response rate in other tumor types, such as melanoma and non-small-cell lung cancer, the underlying mechanism is still unknown, although it might be related to immune cells’ reactivity, increasing T-cell infiltration [38,39,40]. 

An ongoing recruiting four-part, phase 1/2 dose-escalation/expansion study is aiming to evaluate the effects of BDC-1001 (immune stimulating antibody conjugate (ISAC), consisting of an anti-HER2 monoclonal antibody conjugated to a TLR 7/8 dual agonist) in combination with/without PD1 inhibitor pembrolizumab in patients with progressive HER2-expressing solid tumors (NCT04278144). Current results demonstrate BDC-1001 to be well tolerated and clinically efficient also in patients previously treated with anti-HER2 therapy; however, the safety and efficacy of combining with a PD1 inhibitor is yet to be studied [6,41], although the data on PD-L1 expression in CRC with its regards to microsatellite instability remain controversial [38]. Some authors advocate that HER2-targeted therapies may favorably be combined with other emerging therapeutic strategies for advanced CRC, including immune checkpoint inhibitors, increasing the tailored therapeutic approach [42,43].

## 3. Spectrum and Heterogeneity of HER2 Expression in Colorectal Cancer

**Figure 1:**
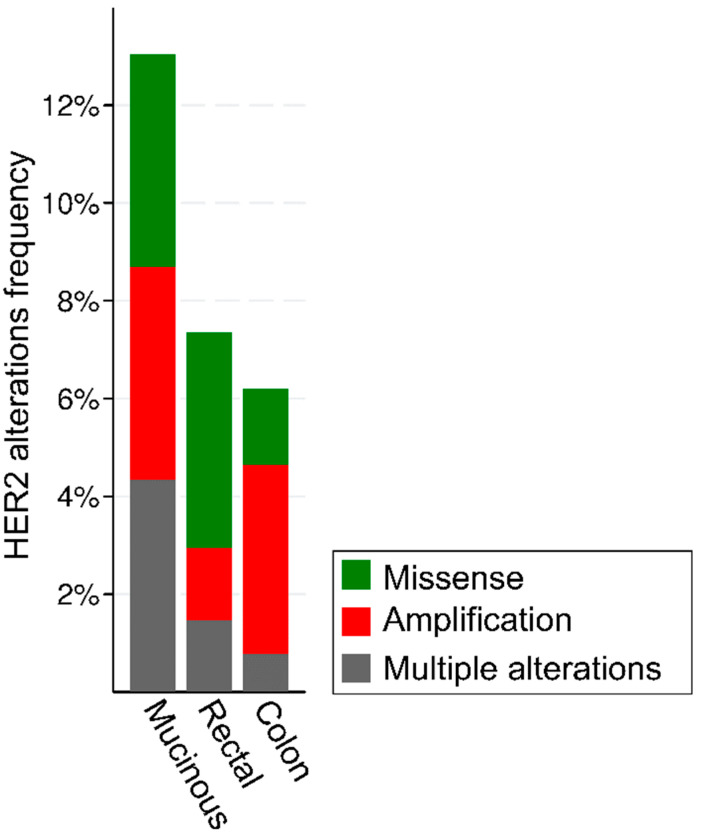
Frequency and types of HER2 alterations in 636 patients with colorectal cancer from the TGCA Firehose Legacy series from https://www.cbioportal.org/, accessed on 11 August 2022.

The analysis of publicly available genomic datasets shows that the frequency of HER2 alterations in CRC ranges from ~6% of colon adenocarcinomas to ~7.5% of rectal carcinomas, with a particularly high frequency in the mucinous histological subtype (Figure 1). Of note, the frequency of alteration types, namely gene amplifications and missense mutations, is heterogeneous based on the anatomical site.

Independent cell growth signaling pathways are activated by several types of *HER2* gain-of-function mutations, including S310F, L755S, V777L, V842I, and L866M. These mutations are clinically actionable in different tumors, as summarized in Table 2.

**Figure 2:**
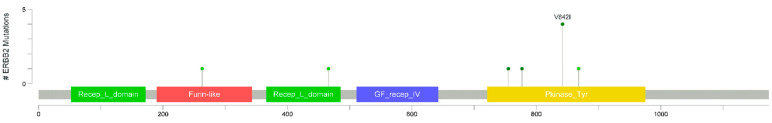
Lollipop plot showing the frequency of HER2 missense mutations in 16 patients with HER2-mutated colorectal cancer from the TGCA Firehose Legacy series from https://www.cbioportal.org/, accessed on 11 August 2022.

These oncogenic mutations cause EGFR antibody resistance in CRC cell lines, and patient-derived xenografts with S310F, L755S, V777L, V842I, and L866M mutations show durable tumor regression when treated with dual HER2-targeted therapy [9]. Of note, position 842 is a hotspot in colorectal cancer, as shown in Figure 2.

The amplification of *HER2* most frequently occurs in the rectum and then other parts of the colon, and it has been associated with acquiring resistance to EGFR-targeted therapies and shorter overall survival compared to *HER2* wild-type CRC [7].

Currently, the expression of HER2 in CRC is evaluated by using IHC and reflex ISH assays. IHC expression is based on the pattern and intensity of membranous reactivity and the percentage of immunoreactive cells. Both circumferential and basolateral/lateral patterns are considered, and the scoring ranges from 0 to 3+ similarly to breast and gastric cancer guidelines [44,45]. Score 3+ is considered positive (intense staining in >10% of tumor cells), and 2+ (weak to moderate staining in >10% of tumor cells) is determined to be equivocal similarly to other cancer types, while 1+ (faint staining in >10% of tumor cells) and 0 (no staining or staining in <10% of tumor cells) are considered negative. Similar to other tumor types, HER2 expression may not be homogenous in all CRC cells. Unlike breast or gastric cancers, HER2 in CRC, detected in the absence of membranous staining, in some reports is defined as the cytoplasmic expression and determined to be an adverse prognostic factor [4,6,14,20,46,47,48]. *HER2* amplification is assessed by fluorescent in situ hybridization (FISH), silver in situ hybridization (SISH), or chromogenic in situ hybridization (*CISH*) counting the *HER2/CEN17* or *HER2/CEP17* signal ratio from 100 nuclei per case The *HER2*/*CEN17* ratio ≥2.0 and *HER2/CEP17* ratio >2.2 (CAP/ASCO guideline 2007) are considered amplified [10,14,48,49]. Comparing breast cancer and CRC, both tumor types exhibit a stronger correlation between HER2 protein expression and *HER2* amplification by FISH and higher intratumor heterogeneity in the case of amplification, dissimilar to gastroesophageal cancer [47,49]. It has been proposed to mold the HER2 testing criteria and interpretation guidelines employed in breast and gastric cancer for HER2 status assessment in CRC [6,49]. However, the HER2 detection and scoring methods for CRC still lack standardization [4,6,14,20,49,50,51]. 

The HERACLES trial suggested both *HER2* overexpression and amplification be accounted as positive predictive markers for anti-HER2 treatment response. Knowing that *HER2* could also represent an important therapeutic target in *KRAS* wild-type metastatic colorectal cancer patients resistant to anti-EGFR treatment, the authors determined the prevalence rate for *HER2* amplification in *KRAS* wild-type samples, which were almost identical (5.1% and 5.2%) [10,14,49,52,53]. Given the presence of *RAS* mutations in CRC, some researchers advocate for the use of next-generation sequencing (NGS) to detect *HER2* amplifications and, thus, refine the selection of metastatic CRC patients, who may be candidates for anti-EGFR therapy [11,13,47,53,54].

## 4. Genetics and Actionable Mutations in HER2-Positive Colorectal Cancer 

**Figure 3:**
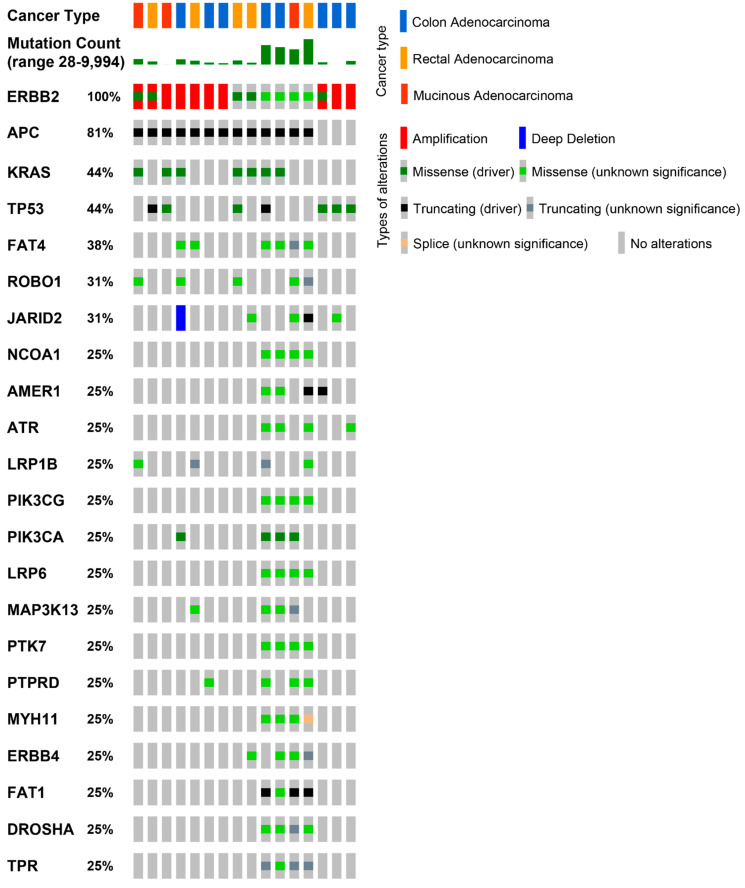
Recurrent genomic alterations in colorectal carcinomas with HER2 alterations. Oncoprint visualization of the most frequently mutated cancer genes in colorectal carcinomas harboring HER2 amplification, mutations, or both types of alterations. TGCA Firehose Legacy series (16 samples) from https://www.cbioportal.org/, accessed on 11 August 2022.

To achieve the most precise patient stratification and to choose the best therapeutic strategy, pathologists and oncologists are actively conducting remarkable work to deepen the clinical and molecular landscape of *HER2*-positive CRC [43]. From a molecular perspective, this is of major interest, considering that due to homo- or hetero-dimerization with other EGFR family members, *HER2* causes transphosphorylation of the intracytoplasmic tyrosine kinase domain, which in turn activates several downstream signal transduction pathways, including *RAS/RAF/ERK*, *PIK3K/AKT/mTOR*, and *JAK/STAT3* [17]. Thus, assessing co-occurring alterations in *HER2* and these pathways may unravel the mechanisms of both primary and acquired resistance to *HER2* inhibition. In breast cancer, among the most frequent alterations that can confer resistance to anti-*HER2* drugs are *PI3KCA* mutations and/or PTEN loss [55,56]. Importantly, the study published by Loree et al. sought to determine the molecular landscape of *HER2/ERBB3*-mutated CRC in three different cohorts composed of a total of more than 2500 patients [57]. In terms of co-occurring alterations, the authors reported a strong association between *PI3KCA* and *HER2* mutations, suggesting that concomitant genetic alterations in the former may represent a second hit to oncogenic signaling [57]. Moreover, they showed that microsatellite instability (MSI) can be correlated with *HER2/ERBB3* mutations potentially explained by the hypermutator phenotype that characterizes these tumors [58,59,60,61]. These findings have been verified by an even larger study in which almost 9000 CRC cases were analyzed by comprehensive genomic profiling for alterations in 315 cancer-related genes, tumor mutational burden, and MSI [50]. Specifically, the authors demonstrated that *HER2* short variant mutations had higher mutation frequencies in the PI3K, mismatch repair (MMR), and Wnt pathways. Regarding the data on co-occurring MSI/MMR-affected function and mutations in *HER2/ERBB3*, the recent study published by Qiu and colleagues reported that cases of HER2-mutated CRC are more likely to harbor an MSI-high status in comparison with the HER2 amplified ones [36]. On the other hand, there is still a debate related to the data on the presence of mutations in the RTK/RAS pathway in patients with *HER2*-positive CRC. While some studies demonstrated that most mutations in *KRAS*, *NRAS*, *BRAF, HER2,* and *ERBB3* are mutually exclusive [62], others reported that *KRAS* alterations have a higher probability to cooccur with short variant mutations in *HER2* than amplification [50]. Considering the clinical importance of co-occurring alterations in the setting of *HER2*-positive CRC, unraveling their prevalence and functional impact represents a crucial need to better establish the optimal targeting of this pathway. As shown in Figure 3, among HER2-altered CRCs (either HER2-mutated, amplified, or both), the most recurrently altered gene is adenomatous polyposis coli (*APC*), which is invariably targeted by truncating pathogenic mutations. Other highly recurrent mutations involve *KRAS* (44%); *TP53* (44%); and members of the PIK3 family, such as *PIK3CA* and *PIK3CG* (25%). 

## 5. Perspectives for Individualized Therapeutic Schemes

An open issue in the treatment of *HER2*-positive mCRC is the continuation of *HER2* blockade in patients developing resistance to anti-*HER2* therapy. The HERACLES RESCUE trial aims to investigate the activity of TDM-1 in patients already treated with lapatinib + trastuzumab in the HERACLES-A trial. In preclinical models of xenograft derived from these patients with acquired resistance to trastuzumab + lapatinib and subsequent exposure to TDM-1, substantial tumor regression was detected [63]. Multiple studies are evaluating the combination of checkpoint inhibitors with anti-*HER2* agents. BDC-1001 is a novel immune-stimulating antibody conjugate (ISAC), consisting of a trastuzumab biosimilar chemically conjugated to a toll-like receptor 7/8 agonist. This compound combines the precision of a tumor-targeting antibody with the therapeutic effect of an immune-modulator and activates at the same time both the innate and adaptive immune responses. A phase I-II study is evaluating BDC-1001 alone and in combination with pembrolizumab in *HER2*-positive tumors. Preliminary results showed a clinical benefit in three out of five mCRC patients treated with one PR and two SD in patients with microsatellite-stable tumors [41,64]. Another ongoing study is evaluating BDC-1001 as a single agent or in combination with nivolumab (NCT04278144). Another ISAC, SBT6050, is being tested alone and in combination with PD-1 inhibitors pembrolizumab and cemiplimab in the same subset of patients (NCT04460456). Zanidatamab (ZW25) is a *HER2*-bispecific antibody targeting simultaneously the juxtamembrane domain (ECD4) and the dimerization domain (ECD2) of *HER2* [65]. A phase II, open-label, two-part, first-line study is testing the combination of chemotherapy and ZW25 in different primary tumors including mCRC (NCT03929666). Different cancer vaccines including *HER2* peptides recognized by T-lymphocytes have been developed so far, such as the TAEK-VAC-HerBy vaccine that is being tested in a phase I study including HER2-positive tumors (NCT04246671). Moreover, different trials are testing chimeric antigen receptor (CAR) T-cells targeting *HER2*, such as *HER2*-specific CAR T-cells in combination with an intra-tumor injection of CAdVEC, an oncolytic adenovirus that is designed to help the immune system, including *HER2*-specific CAR T-cells, react to the tumor (NCT03740256) [66,67]. Natural killer (NK) cells and chimeric antigen receptor macrophages have also been developed [17]. For instance, ACE 1702 is an NK cell product targeting *HER2* expressing solid tumors. A phase I study is ongoing with patients receiving treatment with cyclophosphamide and fludarabine followed by ACE1702 (NCT04319757). In addition, CT-0508 adenoviral transduced autologous macrophages engineered to contain an anti-*HER2* CAR are also under testing in a phase I study of subjects with *HER2* overexpressing solid tumors (NCT04660929).

## 6. Conclusions

HER2 is an established therapeutic target with the continuous evolution of specific anti-HER2 therapeutic agents (monoclonal antibodies, antibody-drug conjugates, and bispecific antibodies targeting) [4,5,6]. Recent data indicate that *HER2* mutations may be successfully targeted by anti-HER2 therapies in various cancer types [4,5]. Clinical trials already report impressive results in overall breast and gastric cancer survival improvement. The trials on HER2-targeted therapies’ application in other solid tumor types are still ongoing [4,68]. *HER2* gene amplification and protein overexpression are identified in about 6% of CRC patients and may be successfully evaluated both by IHC and ISH with high concordance rates, giving these patients new treatment opportunities by making them potential candidates for anti-HER2 therapy [6,10,12,14,50]. Available data in HER2-positive metastatic CRC provide future directions for biomarker-driven research and clinical trials, where the main strategies focus on targeting HER2 as the primary carcinogenesis driver and attempt to overcome other target therapies’ resistance, mediated by *HER2* alterations [6,10,12,14,50].

The prognostic role of HER2 in metastatic CRC remains unclear, although personalized treatment strategies allow for the overall survival of patients to improve and significantly decrease drug-related toxicity. Common widespread next-generation sequencing may provide additional helpful insights [69,70]. The results of novel clinical trials will likely allow HER2 to become a validated therapeutic target in metastatic CRC [4,5,11,47,71].

## Figures and Tables

**Table 1:** Main phase II studies with HER2-targeted therapies in refractory HER2-positive mCRC.

Reference	Nr. of Patients	HER2 Overexpression (%)	Treatment	ORR (%)	mPFS	mOS
HERACLES-A	32	22 (2+); 78 (3+)	trastuzumab + lapatinib	28	4.7	10
HERACLES-B	31	20 (2+); 80 (3+)	pertuzumab + TDM-1	9.7	4.1	-
MyPathway	57	100	trastuzumab + pertuzumab	32	2.9	11.5
TRIUMPH	18	100	trastuzumab + pertuzumab	35	4	-
TAPUR	28	100	trastuzumab + pertuzumab	14	3.8	-
DESTINY-CRC01	78	A: 68 (3+/2+ IHC+); B: 9 (2+ IHC−); C: 23 (1+)	trastuzumab deruxtecan	A: 45.3; B/C: −	6.3 -	15.5 -
MOUNTAINEER (recruiting)	26	100	trastuzumab + tucatinib	52.2	8.1	18.7
HER2-FUSCC-G (recruiting)	11	100	Trastuzumab + pirotinib	45.5	7.8	14.9

Legend: IHC: immunohistochemistry; mOS: median overall survival; mPFS: median progression-free survival; Nr.: number; ORR: overall response rate.

**Table 2:** Cancer type-specific *HER2* S310F, L755S, V777L, V842I, and L866M alterations that may predict response to a targeted drug and the corresponding OncoKB™ level of evidence assigning their level of clinical actionability. Level 2: Standard care biomarker recommended by the NCCN or other expert panels predictive of response to an FDA-approved drug in this indication. Level 3A: Compelling clinical evidence supports the biomarker as being predictive of response to a drug in this indication but neither biomarker nor drug is standard of care.

Cancer Type	Drug	Level
NSCLC	Ado-Trastuzumab Emtansine	2
NSCLC	Trastuzumab Deruxtecan	2
NSCLC	Neratinib	3A
NSCLC	Trastuzumab + Pertuzumab + Docetaxel	3A
Breast cancer	Neratinib	3A

Legend: NSCLC, non-small cell lung cancer.

## Data Availability

Not applicable.

## Supplementary Materials

The following supporting information can be downloaded at: https://www.mdpi.com/article/10.3390/life12091403/s1, Figure S1: HER2 pathways schematic overview. L, ligand, binds to the of human epidermal growth factor receptor (HeR1/3/4) extracellular domain, stabilizing the active HER2 heterodimers formation; PI3K, phosphoinositide 3-kinase; AKT, protein kinase B; mTOR, mammalian target of rapamycin; SOS, Son of Sevenless protein; RAS, member of signal cell transduction proteins; RAF, proto-oncogene serine/threonine-protein kinase; MEK, mitogen-activated protein kinase kinase enzyme; MAPK, mitogen-activated protein kinase.
