# Supplementary material for: HER2 in Metastatic Colorectal Cancer: Pathology, Somatic Alterations, and Perspectives for Novel Therapeutic Schemes
Source: Life (Basel). 2022 Sep 9;12(9):1403. doi: 10.3390/life12091403 (PMC9502498; doi:10.3390/life12091403)
Supplement: Supplementary file 1 [file life-12-01403-s001.zip › life-1895537-supplementary.pdf]

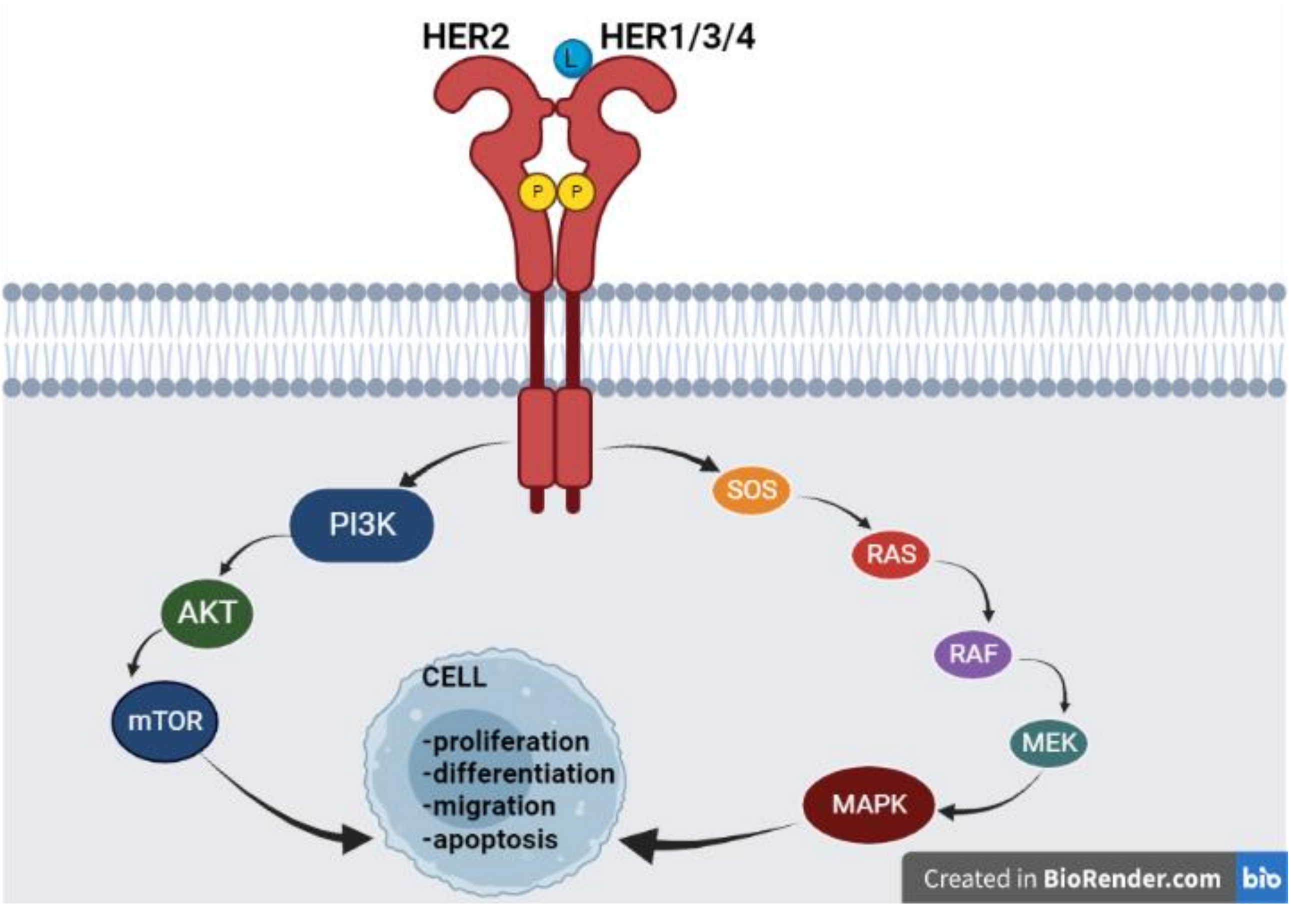

Supplementary Figure S1. HER2 pathways schematic overview. L, ligand, binds to the of human epidermal growth factor receptor (HeR1/3/4) extracellular domain, stabilizing the active HER2 heterodimers formation; PI3K, phosphoinositide 3-kinase; AKT, protein kinase B; mTOR, mammalian target of rapamycin; SOS, Son of Sevenless protein; RAS, member of signal cell transduction proteins; RAF, proto-oncogene serine/threonine-protein kinase; MEK, mitogen-activated protein kinase kinase enzyme; MAPK, mitogen-activated protein kinase.
